# The complete mitochondrial genome of *Neopagetopsis ionah* (Channichthyidae, neopagetopsis) with phylogenetic consideration

**DOI:** 10.1080/23802359.2018.1532350

**Published:** 2018-10-26

**Authors:** Chengde Sang, Shuzhang Liang, Wei Song, Hongliang Huang, Keji Jiang, Xuezhong Chen

**Affiliations:** a School of Ocean, Yantai University, Yantai, Shangdong, China;; b Key Laboratory of Oceanic and Polar Fisheries Ministry of Agriculture, East China Sea Fisheries Research Institute Chinese Academy of Fishery Sciences, Shanghai, China;; c College of Fisheries and Life Sciences, Shanghai Ocean University, Shanghai, China

**Keywords:** *Neopagetopsis ionah*, mito-genome, genome structure, phylogenetic tree

## Abstract

In this study, the complete mitochondrial genome of *Neopagetopsis ionah* was obtained, which was 17,634 bp including two ribosomal RNAs, 13 protein-coding genes, 22 transfer RNAs, and a non-coding control region. In 13 protein-coding genes, there types of initiation codon (ATC, ATG, and GTG) and four types of stop codons (TAA, TAG, TA, and T) were identified. Among the 22 transfer RNAs, eight tRNAs were encoded by L-strand. The length of D-loop was 1519 bp and its contents of A, T, C, and G were 26.9%, 27.6%, 17.5%, and 30%, respectively. The complete mtDNA sequences of *N. ionah* and other 13 species were used to reconstruct the phylogenetic tree suggested that *N. ionah* was closest to some species of Channichthyidae. The study would provide a basic data for further research on population structure, conservation genetics and molecular evolution of *N. ionah*.

*Neopagetopsis ionah* (Channichthyidae, Neopagetopsis), the only known member of its genus, is a species of crocodile icefish found in the Southern Ocean (Circum-Antarctic on the continental shelf and slope) at depths of from 20 to 900 m (Gon and Heemstra 1990). The optimum water temperature is 1 °C. The species grows to a length of 56 cm and adults mainly prey on fishes and krill (Gon and Heemstra 1990). *Neopagetopsis ionah* is the only known member of its genus. So far, no complete mitochondrial sequence information is available. The study is important for the Antarctic ecosystem and further research on genetics and evolution of *N. ionah*.

Adult fish of *N. ionah* was collected from Antarctic (60°21′36″S, 46°41′24″W), it was transported to East China Sea Fisheries Research Institute, Chinese Academy of Fishery Science after freezing at −80 °C. The genomic DNA was extracted from muscle tissues using Animal Genomic DNA Extraction Kit (Tiangen, Beijing, Chian) following the operation manual. The amplifying and sequencing primers were designed according to the sequence of *Chionodraco rastrospinosus* (MF622064) and *Chionodraco hamatus* (KT921282) and sequenced using the Illumina HiSeq2000 platform (Illumina, San Diego, CA).

We obtained the complete mitochondrial sequence of *N. ionah* and submitted it into the Genbank database with an accession number MF596172. This complete mitochondrial genome is 17,634 bp in length, including 13 protein-coding genes, tw rRNA, 22 tRNA, and a control region. The overall nucleotide composition is 25.46% for A, 26.34% for T, 19.01% for C, and 29.19% for G. In 13 protein-coding genes, three kinds of start codons were identified (ATG, ATC, and GTG), ND1 and CO2 were started with GTG and ATC, respectively, whereas others started with ATG. Four types of stop codons (TAA, TAG, TA, and T) were detected. Eight genes (ND6, ND4, ND3, ND2, ND1, CO1, CO3, and Cytb) ended with T. Among the 22 tRNAs, eight tRNAs (tRNA^Pro^, tRNA^Glu^, tRNA^Ser^, tRNA^Tyr^, tRNA^Cys^, tRNA^Asn^, tRNA^Ala^, and tRNA^Gln^) were encoded by L-strand, when others were encoded by H-strand. The length of D-loop was 1519 bp and its overall base composition was 26.9% for A, 27.6% for T, 17.5% for C, and 30% for G, with a high AT content of 54.5%, accord with the structural characteristics of AT rich (Song et al. 2016, Liang et al. 2018). It also has many different lengths of base repeat.

To evaluate the phylogenetic position of *N. ionah*, the phylogenetic tree was reconstructed based on complete mtDNA sequences using the neighbour-joining method in MEGA 5.1 (Kumar et al. 2008) (Figure 1). *Solivomer arenidens* (Myctophiformes, Neoscopelidae), *Neoscopelus macrolepidotus* (Myctophiformes, Neoscopelidae) and *Electrona carlsbergi* (Myctophiformes, Myctophidae) were used as an out-group. The phylogenetic tree showed that *N. ionah* clustered with *C. hamatus*, *C. rastrospinosus* and other fishes in family Channichthyidae. Besides, the out-group formed a big sister branch as well.

**Figure 1.**
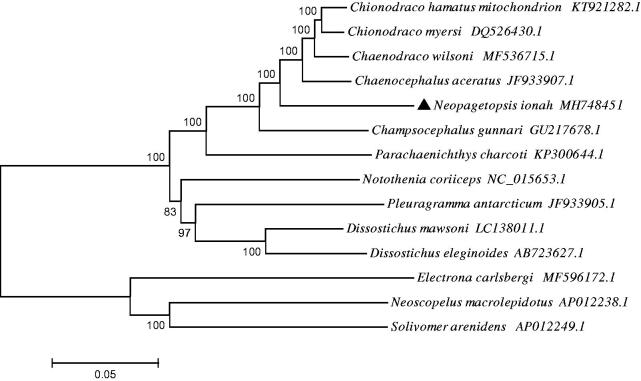
The phylogenetic tree based on complete mtDNA sequences using the neighbour-joining method in MEGA 5.1. *Neopagetopsis ionah* was highlighted with a black triangle.
